# Gamma Activation in Young People with Autism Spectrum Disorders and Typically-Developing Controls When Viewing Emotions on Faces

**DOI:** 10.1371/journal.pone.0041326

**Published:** 2012-07-31

**Authors:** Barry Wright, Ben Alderson-Day, Garreth Prendergast, Sophie Bennett, Jo Jordan, Clare Whitton, Andre Gouws, Nick Jones, Ram Attur, Heather Tomlinson, Gary Green

**Affiliations:** 1 Lime Trees Child, Family and Adolescent Unit, North Yorkshire and York Primary Care Trust, York, United Kingdom; 2 Hull York Medical School, University of York, York, United Kingdom; 3 York Neuroimaging Centre, University of York, York, United Kingdom; University of Bern, Switzerland

## Abstract

**Background:**

Behavioural studies have highlighted irregularities in recognition of facial affect in children and young people with autism spectrum disorders (ASDs). Recent findings from studies utilising electroencephalography (EEG) and magnetoencephalography (MEG) have identified abnormal activation and irregular maintenance of gamma (>30 Hz) range oscillations when ASD individuals attempt basic visual and auditory tasks.

**Methodology/Principal Fndings:**

The pilot study reported here is the first study to use spatial filtering techniques in MEG to explore face processing in children with ASD. We set out to examine theoretical suggestions that gamma activation underlying face processing may be different in a group of children and young people with ASD (n = 13) compared to typically developing (TD) age, gender and IQ matched controls. Beamforming and virtual electrode techniques were used to assess spatially localised induced and evoked activity. While lower-band (3–30 Hz) responses to faces were similar between groups, the ASD gamma response in occipital areas was observed to be largely absent when viewing emotions on faces. Virtual electrode analysis indicated the presence of intact evoked responses but abnormal induced activity in ASD participants.

**Conclusions/Significance:**

These findings lend weight to previous suggestions that specific components of the early visual response to emotional faces is abnormal in ASD. Elucidation of the nature and specificity of these findings is worthy of further research.

## Introduction

Autism spectrum disorders (ASDs) are characterised by difficulties in social and communicative functioning, imagination skill deficits and the presence of repetitive and stereotyped patterns of behaviour [1]. One area of proposed difficulty for individuals with an ASD relates to the recognition of emotions from facial expressions [2]. Reviews of this area show very mixed findings [3], [4]. Whilst intact emotion recognition abilities in ASD have been observed in some studies [5]including identification of complex emotional expressions such as pride and shame [6] many other behavioural studies have reported that children and young people with ASDs are less accurate and take longer than matched controls to identify basic emotions such as happiness and sadness [7]–[9]. Other studies have reported difficulties with recognising specific emotions, such as fear or anger [10]–[12] alongside intact recognition of other basic expressions.

Problems with recognising emotions may result from underlying differences in how information from faces is processed by people with autism spectrum disorders. Neuroimaging studies of ASD face processing have largely utilised functional magnetic resonance imaging (fMRI) techniques to explore this [13], [4]. Studies using fMRI have observed hypoactivation in the fusiform gyrus and amygdala in ASD individuals when viewing faces [14]–[17], although not always [18]–[19], alongside recruitment of non-face areas, including the precuneus, anterior cingulate and cerebellum [20]. In addition, studies examining functional connectivity have documented reduced synchrony between brain areas during face processing in ASD [21]–[24].

These findings (hypoactivation, recruitment of additional cortical regions, and reduced connectivity) may reflect different higher-cognitive processes being applied to relatively intact perceptual information from primary visual areas. That is, faces may be perceived in a typical fashion by people with autism, but not seen as objects that have a particular significance for further social cognitive processing [25], leading to differences in which brain areas are activated. Alternatively, the patterns of network activation seen in previous fMRI studies could result from differences at very early stages of visual processing.

The temporal precision of electrophysiological techniques speaks to this issue by showing the time course of the response to faces in autism [3]. Studies using electroencephalography (EEG) and magnetoencephalography (MEG) have supported the idea that very early visual processing of faces may be abnormal [26]–[28]. In a study using ERP (event-related potential) and dipole analysis, Wong and colleagues [28] observed typical P1, N170 and P2 responses in children with ASD when viewing faces, but dipole analysis revealed slower and weaker responses in the cuneus, fusiform gyrus and medial frontal gyrus. Magnetoencephalography research using dipole analysis in adults with ASD [26] has reported irregular responses to faces but not objects in participants 30–60 ms after stimulus presentation, alongside irregular lateralisation of the face-specific response. The N170 response to faces often seen in typically-developing individuals [29] has also been observed to be delayed in ASD adults and adolescents [30], [27] and weaker in equivalent MEG responses in ASD children [31].

A technique that has the potential to add much to this area is spatial filtering, or “beamforming”, in MEG. Spatial filtering techniques allow for estimation of the time course of neural activity at specified cortical locations [32]–[34] and have recently been use to localise and reconstruct responses to a wide range of sensory and cognitive systems [35]–[39]. Such analyses allow for the examination of early temporal responses to faces, as in previous EEG/MEG research, but also specific localisation of that response to cortical areas identified from fMRI work. The only MEG studies to have looked at face processing in ASD previously [26], [32] did not use spatial filtering approaches such as beamforming to examine emotion recognition.

A further advantage of beamforming in MEG is its ability to extract detailed information on the phase and frequency of neural responses. Abnormalities in the gamma range (30–80 Hz) in particular have been observed in a range of sensory processing studies on autism. In an EEG study of adults with ASD, Grice and colleagues [40] found a typical increase in gamma oscillations to face stimuli, but no modulation of the gamma response compared to controls when the faces were inverted. Other studies have also reported irregular gamma responses [41] including irregular gamma modulation in responses to auditory stimuli for both children [42] and adults with autism [43].

A range of theories have arisen about the role of abnormal gamma activity in ASD. Central among these are its proposed role in related areas of visual feature binding [44], [45] gestalt perception [46] and functional connectivity in autism [47]. Gamma activity in response to a stimulus can be separated into “evoked” or phase-locked activity and “induced” or non phase-locked activity; components that are thought to reflect differing underlying neural processes [44], [48]. Activation of induced activity in particular is thought to be necessary in sensory feature binding, facilitating the combination of separate visual components into a coherent whole [44]. Accordingly, it has been suggested that disruptions in gamma production may underlie the observations of “weak central coherence” or local processing bias in ASD [49], where individual features are processed in preference to or at the expense of more global and holistic forms of processing [50], [51]. Importantly, this could also apply to faces. Faces are complex visual objects that require the perception not only of individual features, but also the combination of those features into a coherent whole. It could be that abnormalities in induced gamma functioning underlie disruptions in the way faces are processed in ASD at a very basic level.

Few studies to date have examined the separate evoked and induced gamma responses of individuals with ASD. In Grice et al [40], participants with autism differed in their later, induced gamma responses to faces, but not their earlier, evoked responses to the same stimuli. In an EEG study run with children with autism, Brown et al [49] reported abnormal induced gamma responses to Kanizsa stimuli (i.e. geometric shapes that require visual binding), but specific data on the evoked response were not reported. In contrast, a similar EEG study by Stroganova et al [52] observed reduced evoked responses to Kanisza stimuli in a group of children with ASD, but they did not present results for induced gamma responses. Studies on auditory gamma responses have more consistently reported reductions in evoked activity and phase-locking across trials in ASD individuals [42], [43], [53], [54], although in some cases this has been observed alongside abnormally increased levels of induced activity [43].

As such, abnormalities in induced gamma activity provide a theoretical basis for problems with face processing in autism, but the exact nature of induced vs evoked gamma responses in autism is still a matter of considerable debate. We wished to explore this issue in a group children and young people with autism spectrum disorders, piloting the use of beamforming in MEG to examine the space, time and frequency response of brain activation when people with ASD are viewing emotions on faces. If gamma abnormalities underlie face processing differences in autism, then other frequency responses to faces, such as those in lower frequencies (e.g. below 30 Hz) should be relatively intact. Furthermore, if they lead to irregular timing and utilisation of face networks, then gamma abnormalities should be present very early in the neural response, and in the primary visual cortical areas. Finally, if visual binding is associated with induced gamma responses, and binding is a problem in ASD face processing, then any gamma abnormalities observed should be specific to the induced response. We predicted that evoked responses, in contrast, would be relatively intact in ASD participants.

## Methods

The study was approved by the York ethics committee (REC reference number: 05/Q1108/61) and by the York Alliance Research and Development Committee (reference: NYY-PO529). Ethics approval was also granted by the York Neuroimaging Centre Ethics Committee. All participants gave written informed consent before taking part in the study. Parents consented on behalf of any minor and this was full written consent.

### Participants

13 participants (10 male) with an autism spectrum disorder (ASD) and 13 typically developing controls (TD) aged 9–18 were recruited from local schools (ASD*_M_* = 181.77 months; TD*_M_* = 188.58 months). All ASD participants had a diagnosis of either high functioning autism or Asperger Syndrome in accordance with ICD-10 Research Diagnostic Criteria [1] and were diagnosed through a multidisciplinary panel that considers all ASD assessments in the local child and adolescent mental health service. The Autism Diagnostic Interview – Revised [55] and Autism Diagnostic Observation Schedule – Generic [56] are part of the assessment protocol. Families of all participants also completed the Autism Spectrum Quotient – Adolescent Version [57]. This verified diagnoses with the ASD group scoring significantly higher on the AQ (AS *M* (*SD)* = 38.62 (6.16); TD *M* (*SD*) = 12.33 (6.47) t = 10.405, *df* = 23, *p*<.001). There was no overlap in scores between the two groups.

Exclusion criteria included the presence of any other neurological or developmental conditions which may affect cognitive processing, e.g. Tourette’s syndrome or, Fragile-X syndrome.

Eleven participants (7 ASD, 4 TD) were recruited on the basis of participation in a previous behavioural study [12]. The remaining participants were recruited through contact with local clinical services and Local Authority groups.

### Matching

ASD and TD participants were matched individually on gender, age (+/− 12 months) and full-scale IQ (+/− 10 points). Full-scale IQ estimates were derived using the Wechsler Abbreviated Scale of Intelligence [58]. All participants completed the WASI in a session with a trained researcher prior to scanning. Although participants were not explicitly matched for handedness, the two groups were very similar (ASD: 11 R, 1 L, 1 ambidextrous; TD: 11 R, 2 L; based on parent reports).

### Behavioural Measures

Prior to scanning, participants completed a basic emotion recognition task using 60 faces from the Ekman-60 Faces stimuli set [59] specifically those displaying expressions of surprise, happiness, sadness, fear, anger and disgust from FEEST (the Facial Expressions of Emotions Stimuli and Tests) [60]. 10 models displaying six emotions each were selected, and every face was shown twice. These were displayed at random for 120 consecutive presentations. Participants were asked to identify the expression on each face via a numbered button press (e.g. 1 = surprise, 2 = happiness *etc*.). Accuracy scores and response times were recorded. Response time outliers over two standard deviations from the group mean were trimmed from the data set and all data were normalised using a log10 transform to address negative skew. The behavioural task was conducted prior to MEG recording to avoid noise from motor responses during scanning.

In addition to the faces task, participants’ AQ scores were used to assess how any neurophysiological differences between groups related to ASD behaviour.

### MEG Task

#### MEG recording

All scanning took place at York Neuroimaging Centre using a 4D Neuroimaging Magnes 3600 Whole Head 248 Channel magnetometer scanner. Head-shape was recorded using the Polhemus Fastrak system. Head-shape digitisation was conducted prior to scanning and checked pre- and post-acquisition to check coil location and head position. MEG data were acquired at a sampling frequency of 678.17 Hz using a high-pass filter of 3 Hz and a low-pass anti-aliasing filter of 200 Hz. Reference channels were used to linearly remove electromagnetic interference from outside the scanner [61] and, post-acquisition, data were DC offset to ensure a zero mean signal on all sensors. Epochs containing visible physiological artefacts (eye-blinks, body movement *etc*.) were manually removed prior to analysis.

#### Materials & procedure

Participants were placed in an upright and seated position for all MEG data acquisition. Following head-shape digitisation, participants were given 2–3 minutes to relax and acclimatise to the scanner. Testing stimuli were presented by projection onto a screen positioned approximately 1 metre away from the participant. The images subtended a visual angle of 16×10 degrees (h×w).

Participants began the session by viewing a short set of practice images, depicting the same faces from the Ekman set as those seen in the behavioural task [59]. Participants were asked to view the faces and think about each emotion as they viewed it. A passive viewing paradigm was chosen in order to minimise potential noise from muscle movements in response or decision-making. Following the practice phase, whole-head MEG data were acquired while participants viewed a randomised set of 600 face images (60 face images presented 10 times each). Each face image was presented for 1000 ms, followed by a fixation cross for another 1000 ms. This created an overall testing epoch of 2000 ms which contained “active” and “passive” viewing windows. Image presentation was broken up into 5 blocks of 60 epochs, with 10 seconds break periods in between. Participants were videoed during scanning to ensure that they were awake at all times and attending to the faces. No quantitative information was derived from this data, but a researcher monitored the live video feed during scanning and recommended a rescan should any participant clearly stop attending to the stimuli.

### MRI Recording and Co-registration

Structural data were acquired using a GE 3.0T Signa Excite HDx MRI scanner. A T1 structural image was acquired for each participant using a 3D FSPGR (Sagittal Isotropic 3D Fast Spoiled Gradient Recall Echo - structural T1 weighted scan). The following acquisition parameters were used: Matrix size: 256×256×176, FOV: 290×290×176, Slice thickness: 1.13×1.13×1.0 mm, TR 8.03, TE 3.07, Flip angle 20′′, PSD: efgre3d. MEG and head-shape data were then co-registered to each individual’s MRI structural data to create a whole-head brain map for each participant. Co-registration was based on a method developed by Kozinska et al, [62]. The Montreal Neurological Institute (MNI) standard brain was then used for source localisation in each group. Coregistrations were individually checked prior to beamforming analysis and for each virtual electrode placement. To perform the beamforming analysis, an isotropic grid (5 mm spacing) was placed in the standard brain. This grid was then warped into the brain of each individual using their specific linear transform which describes the relationship between their T1 image and the standard brain. This process ensures that the analysis in each participant has the same number of grid points and each of these locations is in a comparable region across individuals.

### MEG Analysis

#### Beamforming

Beamforming is a spatial filtering approach that uses a weighted sum of the sensor data in order to generate an estimate of the neural time-course of activity. Typically this analysis takes place at many thousands of locations throughout the head. A power estimate is computed for both an “active” and “passive” period of activity (i.e. during stimulus presentation and rest) and a t-test can be applied to these power estimates to reveal regions of the volume in which a stimulus-related change in power was seen [33].

A minimum variance beamformer was used to calculate neural activity across a 5 mm isotropic grid [32], [34]. Details of the exact implementation can be found in Hymers et al [63]. Power estimates for active and passive windows were compared using t-tests to indicate regions which significantly changed [64]. Group images were derived by converting t-scores to z-scores for each voxel in each individual. A one-sample t-statistic was then used to identify volumetric locations which showed consistent stimulus-related power changes across all individuals in conjunction with non-parametric permutation thresholding [65]. The power of using non-parametric permutation testing is that no assumptions are made regarding the underlying statistical distribution and the data are used to empirically characterise the null hypothesis [66]. Maximum statistics were used to account for the multiple comparisons problem [67]. This exact method of beamformer analysis has been described in greater detail in a recent study on face processing in neurotypical adults [35].

Beamforming was conducted across frequency ranges to examine responses in the gamma band (30–80 Hz) and compare them with a “control” lower band (3–30 Hz). The passive epoch window was defined as −300 ms to −100 ms pre-stimulus onset. The screen showed a fixation cross over this time course. This was compared to three 200 ms-long active viewing windows of 50–250 ms, 250–450 ms and 450–650 ms post-stimulus (responses after 650 ms were too diffuse to analyse). Three 200 ms windows were chosen to show the progression of neural activity changes following stimulus onset; windows shorter than 200 ms were not selected as they reduce the possibility of showing low frequency changes (<10 Hz) in oscillatory activity.

#### Virtual electrodes

The spatial filter reconstruction is typically reduced to a single number to quantify the response at many thousands of points within the volume. Once this volumetric analysis has identified regions which respond to the stimulus, the same spatial filter can be used to reconstruct the time series of electrical activity at specific points in the head. These reconstructions can also be termed virtual electrodes (VEs). At the location of interest the spatial filter is constructed in the same way as when the volumetric analysis is performed using the LCMV beamformer. As only one point is now being considered it is unnecessary to reduce the dynamic signal to a single value, and so the entire trace can be recovered. A VE is therefore a weighted-sum of the sensor data that estimates the time-course of electrical activity via a spatial filter.

VEs were placed in three ways. First, a VE was placed based on the maxima derived from a between-groups whole-head beamforming analysis; that is, the peak area of difference between ASD and TD participants when viewing the stimuli. Second, to provide more information on the response within each group, a VE was placed based on the mean position of significant maxima identified in the whole-head beamforming. Finally, a VE was placed in the right fusiform gyrus (MNI mm = 32, −57, −3) due to its putative role in face processing [68]. The placement of this VE in MNI space was based on the findings of previous research on face processing in ASD individuals [69], [70]. Virtual electrodes were placed for each individual by transforming the standardised MNI co-ordinate into the co-ordinate space of each individual using the same linear transform that was used when warping the isotropic grid.

Time frequency plots were then used to compare power differences in active and passive windows for each virtual electrode. The reconstruction estimates the neural time-course at each location for each epoch. These epochs can be averaged in time and then a time-frequency analysis performed to focus on phase-locked, evoked activity. A time-frequency analysis can also be performed on each epoch, and this estimate of power can be averaged to provide an analysis of non-phase-locked, induced information.

A Stockwell transform was used to generate time-frequency estimates [71] and non-parametric permutation statistics were used to derive threshold estimates for the time frequency space [72]. This involves treating the time-frequency bins independently and employing a similar permutation scheme to the one-sampled t-test carried out at the group level when considering the whole volume. The first-level statistics are computed by performing a t-test between the 200 ms active and passive windows in each individual. This produces a t-value at each time-frequency location for each individual. The observed t-statistic is computed across these t-maps for each time-frequency location using a one-sampled t-test. Subsequently, over a series of permutations, a random number of participants have the t-values in their first level t-maps inverted in polarity. At locations where there is little genuine response this will have little effect and the permuted value will be similar to the observed value. Where a genuine effect exists there will be a clear difference between the permuted and observed t-value. Maximum statistics are again used to correct for multiple comparisons.

The use of active vs passive contrasts within individual participants lends itself to within-groups analysis; that is, separate, detailed analyses of the neural response in ASD and TD participants, which can then be compared visually. For a recent example of this in EEG, see [53]. However, between-groups contrasts can provide more immediate information on the main differences between ASD and TD participants. To accommodate both, the first beamforming analysis presented here contrasted ASD and TD participants by statistically comparing pairs of time-windows for each group (e.g. ASD-Active vs TD-Active, 50 ms to 250 ms; ASD-Passive vs TD-Passive; 300 permutations per t-test). This produced t-maps for each time window, indicating voxels that statistically differed in their level of power between-groups. Then, to show the significant voxels that could be considered “event-related”, the t-map for passive differences (representing a baseline) was subtracted from a t-map of active differences. As this involved the combination of two different statistical contrasts (active vs active and passive vs passive), a conservative threshold of p<.001 was chosen for indication of significant voxels. The cut-off t-value for this level of significance was based on whichever of the two tests required a *higher* value to achieve p<.001.

Beamforming and virtual electrode analysis was conducted first across all six emotions combined, Following this, beamforming data was analysed for each specific emotion. Virtual electrode analysis for specific emotions was not conducted due to the reduced signal-to-noise ratio involved in analysing each emotion separately. (Whereas the combined VE analysis was conducted on all 300 epochs, specific emotions could only provide a maximum of 50 epochs each).

## Results

The scanning data for one control participant could not be used due to medical concerns that arose from their structural MRI scan. As such, any data presented below refers to only those participants who completed the study (13 ASD/12 TD). No participants in either group required a rescan due to excessive movement or loss of attention to the task stimuli.

### Behavioural Data

Table 1 displays means for age, IQ and AQ scores for the two groups. T-tests indicated that they did not significantly differ on age, full-scale IQ or any subscale IQ scores, even with the exclusion of the 13th control participant (all *p*-values >.120).

**Table 1:** Age, Full-scale and subscale IQ scores for ASD and control participants.

	ASD (*n* = 13)	TD (*n* = 12)		
	*M*	*SD*	*M*	*SD*
Age (months)	181.77	33.92	188.58	24.83
Full-Scale IQ	109.23	15.15	114.83	12.27
Verbal IQ	111.77	19.11	115.17	13.07
Non-verbal IQ	104.00	13.98	111.92	13.96
AQ	38.62^a^	6.16	12.33	6.47

a *p*<.001.

**Figure 1:**
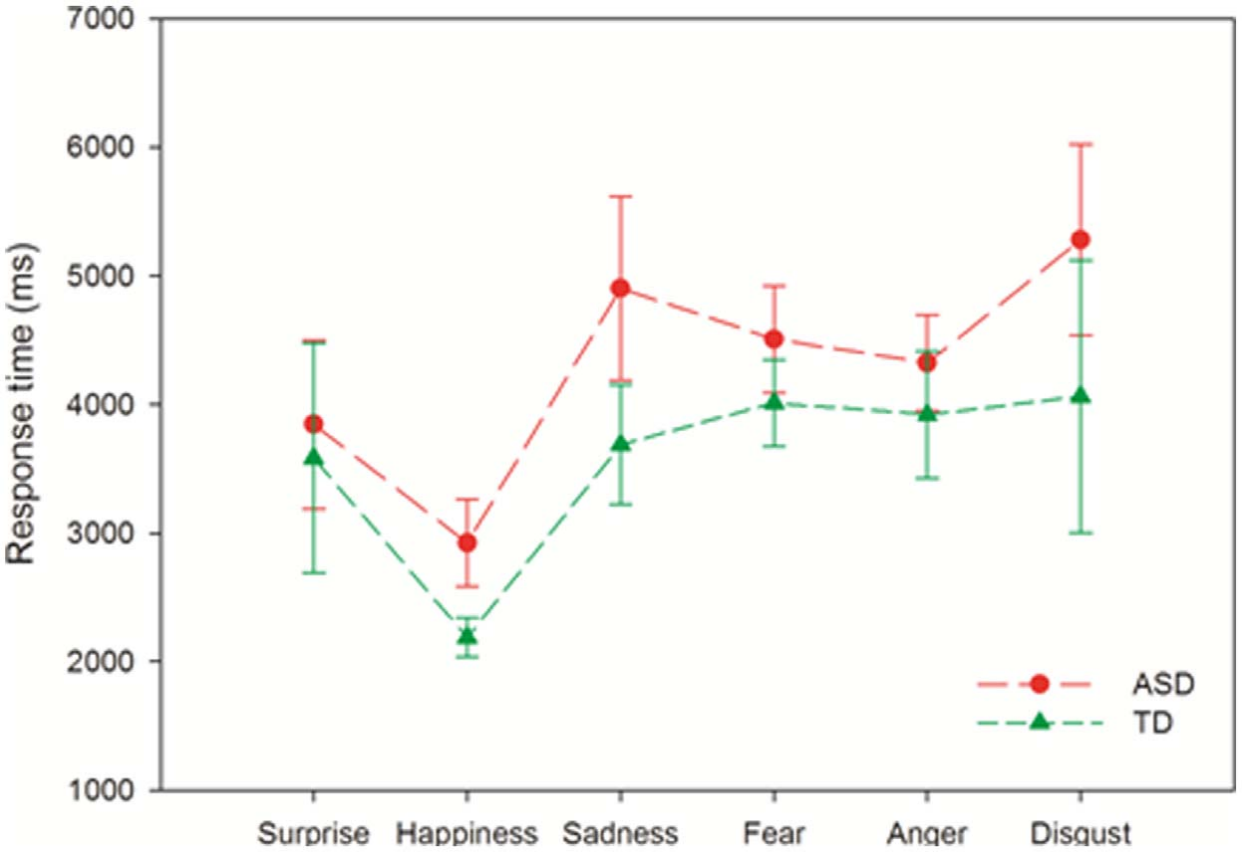
Response times for identifying specific emotions in ASD (red) and control (green) participants.

A mixed analysis of variance was used to compare accuracy scores on the faces task between the two groups and across the different emotion categories. Accuracy scores were highest in both groups when identifying happy faces and lowest when identifying fearful and disgust faces, but no significant main effect of group was observed (*F* (1,23) = 1.050, *p* = .316, eta^2^_p_ = .044). However, there were clear differences in response times. Figure 1 shows that controls were quicker overall to respond to faces (*F* (1, 23) = 4.503, *p* = .045, eta^2^_p_ = .164) but also faster on certain emotion categories (group*emotion interaction effect: *F* (5,115) = 3.312, *p* = .008, eta^2^_p_ = .126). Post-hoc t-tests with a Bonferroni-adjusted alpha-value of *p*<.01 indicated that controls were specifically faster in identifying disgusted faces (*t* = 2.845, *df* = 23, *p* = .009). Mean group differences approaching significance were also observed for happy faces (*t* = 2.279, *df* = 17.765, *p* = .035) and sad faces (*t* = 2.043, *df* = 23, *p* = .053).

### Beamforming

#### Between groups

Three 200 ms windows were analysed: 50–250 ms, 250–450 ms and 450–650 ms post stimulus onset. Analysis of artefact rejection rates indicated no significant differences between the two groups (*t* = −0.161, *df* = 23, *p* = .874) indicating similar levels of signal-to-noise ratio for ASD and TD participants.

**Figure 2:**
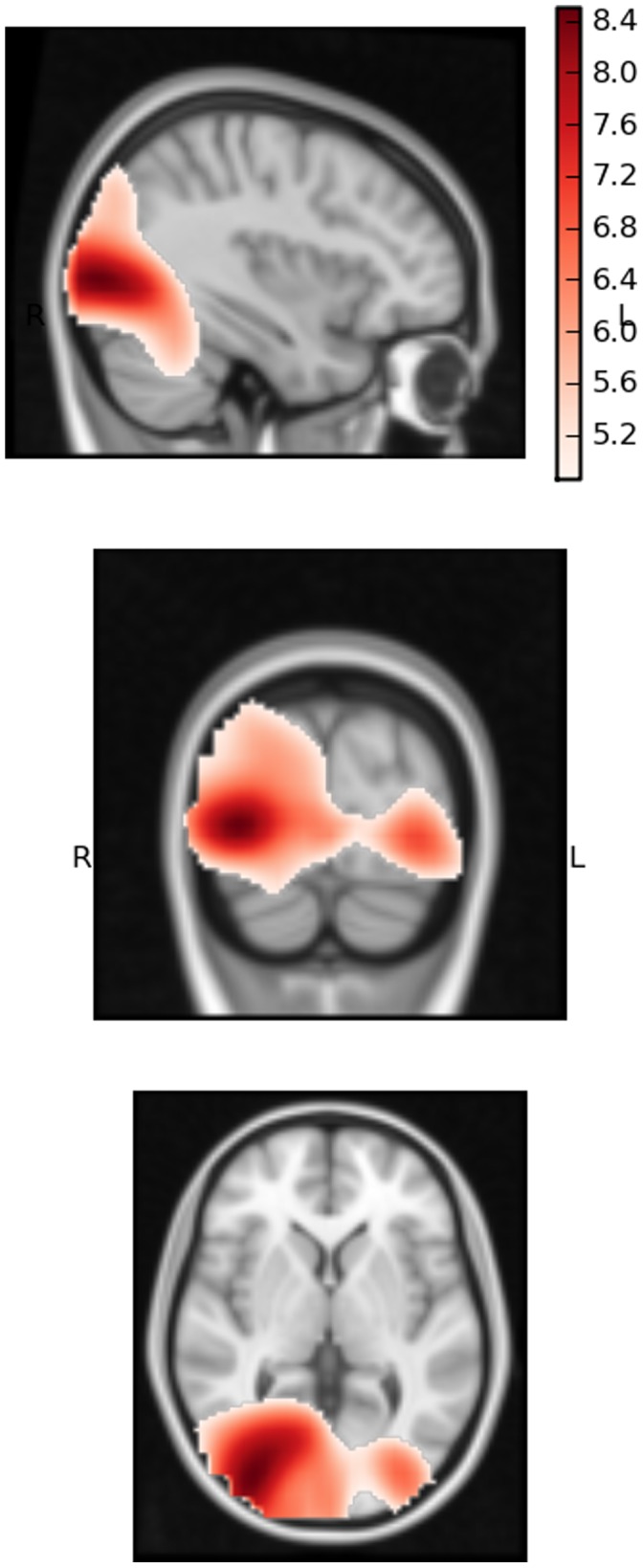
Significant differences in gamma band (30–80 **Hz) response, 250–450** **ms, between ASD and TD participants.** Red  =  TD > ASD; Blue  =  ASD > TD; p<.001 for all highlighted voxels. Slice shown MNI: −36, −86, 2 (voxel of maximum difference).

In the gamma-band analyses significant group differences were observed in right occipital areas in each of the time-windows analysed, with TD participants producing significantly greater levels of power than ASD participants. The peak difference (t = 8.506, p<.001) was observed in a voxel in the inferior division of the right lateral occipital cortex (MNI 36, −86, 2) between 250 and 450 ms (see figure 2). Significantly greater gamma power in TD participants was evident across the right occipital cortex, extending anterior to occipital-fusiform areas, medially to intracalcarine cortex and posterior to the occipital pole. Although slightly weaker (t = 7.0156) and much less widespread, this effect was also seen in the corresponding area of the left lateral occipital cortex (MNI −34, −86, 2), suggesting a bilateral response. No areas showed significantly greater responses for ASD participants.

In the lower band analysis (3–30 Hz) TD participants again showed significantly greater power than ASD participants in all three windows, peaking in the 250–450 ms window. A bilateral response was evident across posterior parts of the occipital lobe, with the maximum response evident in the right occipital pole (t = −5.43, p<.001; MNI: 0, −96, −6).

In summary, the two groups significantly differed in the strength of their gamma and lower-band responses. In gamma the peak difference was observed in right lateral occipital areas. The peak lower-band difference was situated in the right occipital pole.

#### Within groups

Active vs passive contrasts were then run within each group to examine the response to faces in more detail. The significant minima and maxima observed during beamforming are displayed in tables 2 (ASD) and 3 (TD). In ASD participants significant responses in the gamma range were only observable in the 250–450 ms window. Compared to the passive window, significant decreases were evident in the left supramarginal gyrus and left precentral gyrus (see table 2). No significantly changing voxels were evident in the gamma range in occipital areas in ASD participants, suggesting that faces failed to drive a statistically distinct visual gamma response in this group.

**Table 2:** ASD whole-head beamforming.

Band	Window	x	y	z	Region^a^	Max./min. *t*-value
Upper	50–250 ms					
(30–80 Hz)						
		*No sig.voxels observed.*				
	250–450 ms	−66	−40	34	L-SMG	−6.03
		−56	0	28	L-PCG	−5.97
			–		–	–
			–		–	–
			–		–	–
	450–650 ms					
		*No sig. voxels observed.*				
Lower	50–250 ms					
(3–30 Hz)						
		*No sig. voxels observed.*				
	250–450 ms	14	−76	8	R-ICC	−7.29
		−16	−90	8	L-OP/WM	−6.95
		−16	−86	4	L-LOC	−6.29
			–		–	–
			–		–	–
	450–650 ms	44	−70	48	R-LOC	−8.14
		20	−80	−12	R-OFG	−8.01
		34	−96	8	R-OP	−7.60
		10	−76	34	R-CC/PC	−7.43
		14	−86	−34	R-OP/LOC	−7.39

The top five significant maxima/minima in each time window are displayed. All reported voxels indicated significant changes from the passive window at *p*<.05 or below. *X*, *Y* and *Z* reflect MNI co-ordinates. Anatomical labels were based on the Harvard Cortical and Subcortical atlases available in *FSLView* imaging software.

a L-  =  left, R-  =  right, CC  =  cuneate cortex, ICC  =  intracalcarine cortex, LOC  =  lateral occipital cortex, OFG  =  occipital-fusiform gyrus, OP  =  occipital pole, PC  =  precuneal cortex, PCG  =  precentral gyrus, SMG  =  supramarginal gyrus, WM  =  white matter.

**Table 3:** Control whole-head beamforming.

Band	Window	x	y	z	Region^a^	Max./min. *t*-value
Upper	50–250 ms	44	−70	−26	R-LOC	9.97
(30–80 Hz)		−10	−90	−12	L-LG	8.74
		−46	−66	−26	L-OFG	5.58
			–		–	–
			–		–	–
	250–450 ms	30	−70	−16	R-OFG	12.13
		40	−60	−32	(Stem)	10.40
		−10	−80	−16	L-LG/OFG	9.85
		20	−106	−2	R-OP	7.09
			–		–	–
	450–650 ms	−16	−80	−26	L-OFG	8.35
		−20	−106	−6	L-OFG	7.80
		40	−56	−32	(Stem)	5.89
		44	−80	−32	(Stem)	5.81
		30	−100	−6	R-OP	5.75
Lower	50–250 ms					
(3–30 Hz)						
		*No sig. voxels observed.*				
	250–450 ms	54	−70	−12	R-LOC	−8.50
		−40	−86	−6	L-LOC	−8.03
		40	−96	4	R-OP	−7.17
		−20	−90	24	L-OP	−6.16
			–		–	–
	450–650 ms	50	−70	−22	R-LOC	−10.97
		−20	−70	14	L-ICC/SCC	−9.16
		40	−96	4	R-OP	−8.19
		−30	−96	24	L-OP	−7.90
		−46	−80	−2	L-LOC	−7.76

The top five significant maxima/minima in each time window are displayed. All reported voxels indicated significant changes from the passive window at *p*<.05 or below.

a L- = left, R- = right, ICC = intracalcarine cortex, LG = lingual gyrus, LOC = lateral occipital cortex, OFG = occipital-fusiform gyrus, OP = occipital pole, SCC = supracalcarine cortex.

**Figure 3:**
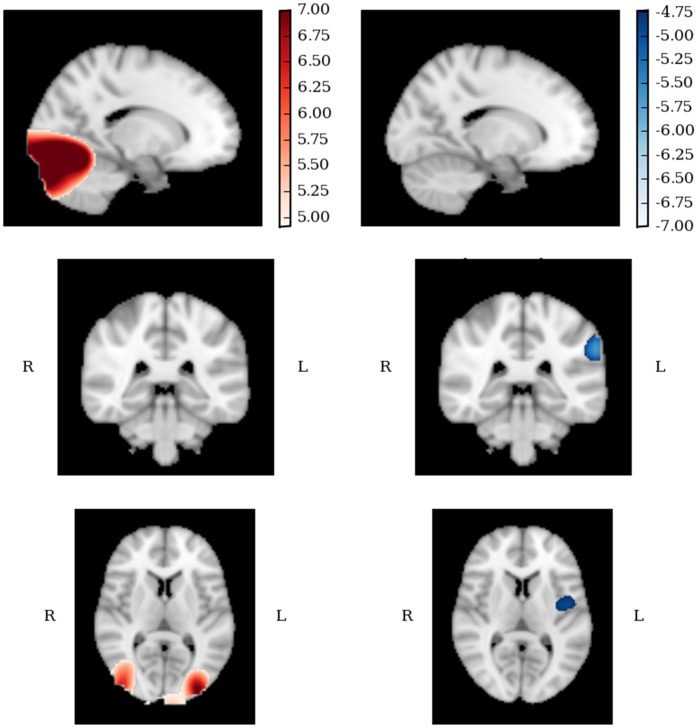
The gamma band response (30–80 Hz), 250–450 **ms, in TD (left) and ASD (right) participants.** Red  =  maxima, blue  =  minima. P<.05 for all highlighted voxels. Slice shown MNI: −16, −56, 8.

Strong bilateral gamma activation was evident in controls, peaking significantly in right lateral occipital cortex, left lingual gyrus and left occipital-fusiform gyrus in the first 250 ms. Following this, increases in power were also evident in right occipital-fusiform gyrus (250–450 ms) and occipital poles bilaterally (250–650 ms) as shown in figure 3. No significant decreases in power were evident at any time.

#### Lower band analysis (3–30 Hz)

**Figure 4:**
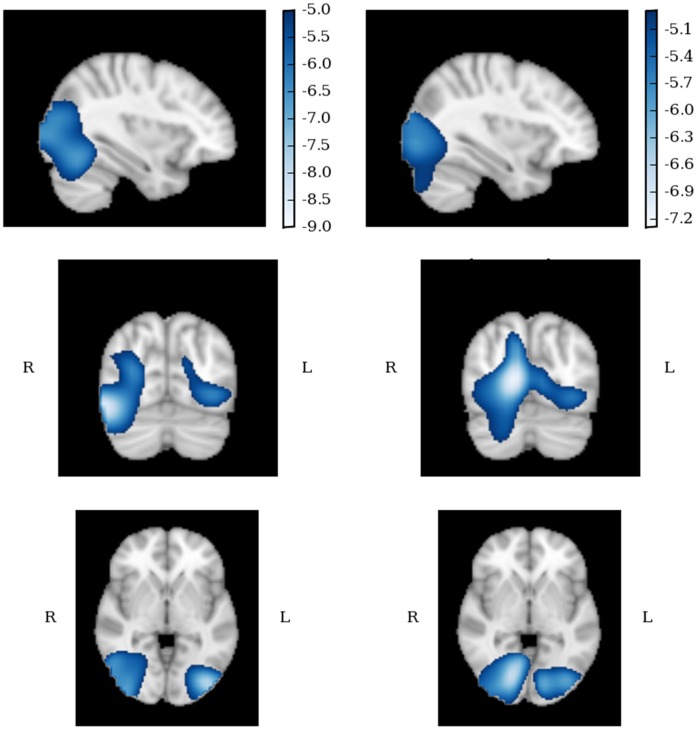
The lower band response (3–30 Hz), in TD (left) and ASD (right) participants. Red  =  maxima, blue  =  minima p<.05 for all highlighted voxels. Slice shown MNI: 32, −72, −2.

Activation in the lower band was similar for both groups across the three time windows. No significant changes were evident for either group in the initial 50–250 ms response. In the second time window both groups showed significant reductions in power in occipital pole and lateral occipital areas, which continued up to 650 ms, although the focus of peak responses for each group appeared to differ. As figure 4 shows, between 250–450 ms the response for ASD participants was spread to medial areas such as intracalcarine cortex, while the control response centred specifically on lateral occipital areas.

#### Summary of beamforming

Large group differences were seen in the gamma band. A strong gamma response was observed in controls in visual areas but was absent in ASD participants (see figure 3), as suggested by the between-groups response. Although the two groups differed in the lower-band between-groups analysis, the within-group analysis indicates that their lower-band responses were actually fairly similar. Both groups showed significant reductions in power in visual areas, the significant group difference highlighting that controls did this *to a lesser degree* than ASD participants. This suggests that abnormalities in the ASD response in the gamma range were accompanied by potential over-reductions of lower-band power.

### Virtual Electrodes and Time-Frequency Analysis

The first virtual electrode was placed in the right lateral occipital cortex (36, −86, 2) where the peak group difference was observed for the gamma-band response. Peak areas were also identified during the within-groups whole-head beamforming. A virtual electrode was seeded in the left occipital pole (−20, −92, 16), and a VE was also placed at a theory-driven site in the right fusiform gyrus (32, −57, −3).

#### Right lateral occipital cortex

**Figure 5:**
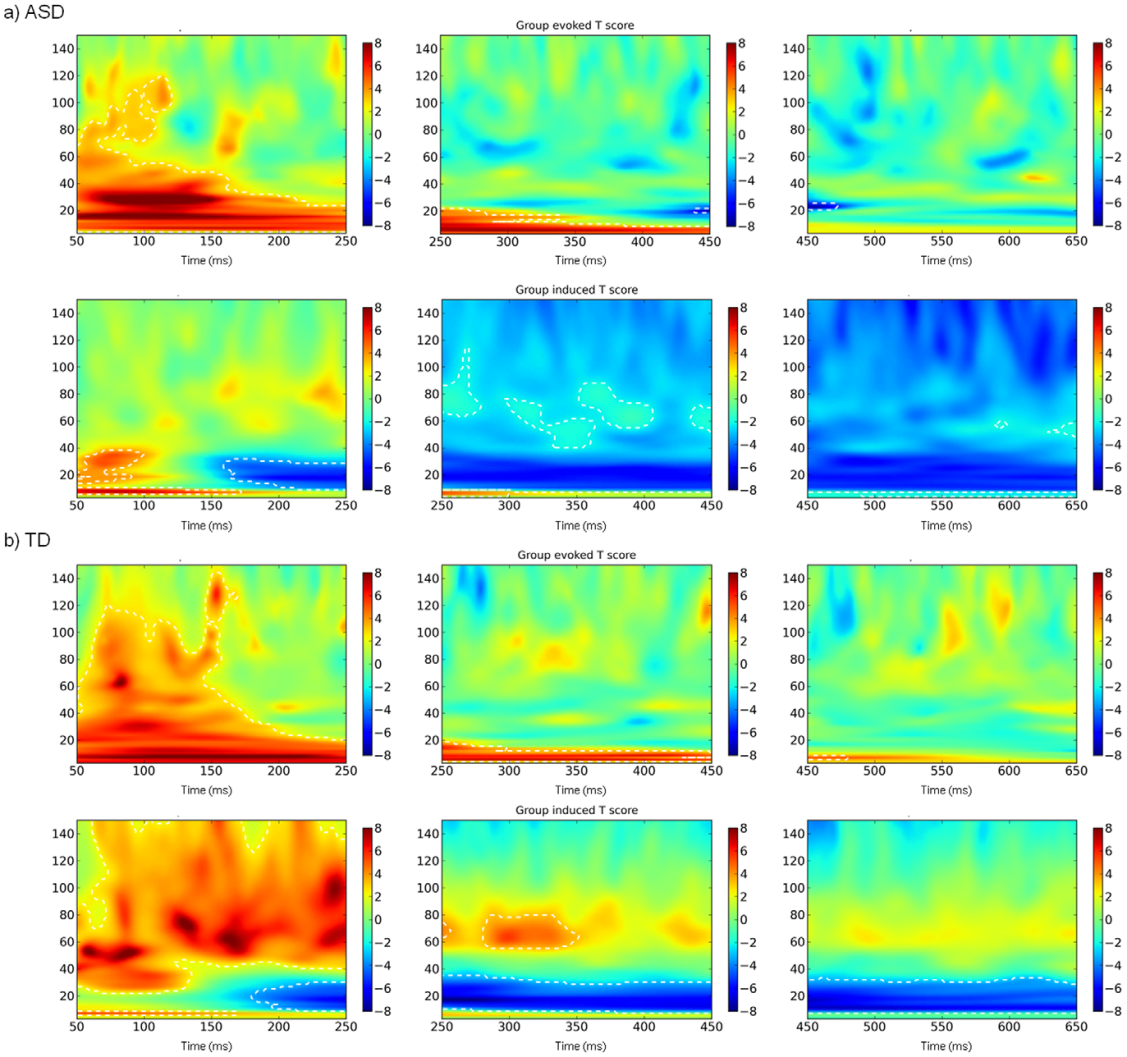
Virtual electrode responses in the right lateral occipital cortex, superior division (R-LOP; 36, −86, 2) for ASD (a) and TD (b) participants. Evoked responses are displayed in the upper row of each figure; induced responses are displayed in the lower row. All responses indicate within subjects changes from baseline; changes significant at p<.05 level are indicated within dotted lines.

Figure 5 displays the evoked (phase locked) and induced (non-phase locked) responses for ASD (top) and TD (bottom) participants when a virtual electrode was placed at this site. Significant evoked responses are evident in both groups in the first 50–100 ms, but after this no significant phase-locked responses were recorded.

In the induced activity, both groups appear to follow their evoked responses with a period of decreasing lower-band activity (10–30 Hz) from 150 ms onwards. However, while ASD participants also show significant decreases in induced power at higher frequencies (>30 Hz) from 250 ms onwards, TD participants show significant increases in induced gamma power that are evident in the 50–250 and 250–450 ms windows.

#### Left occipital pole

**Figure 6:**
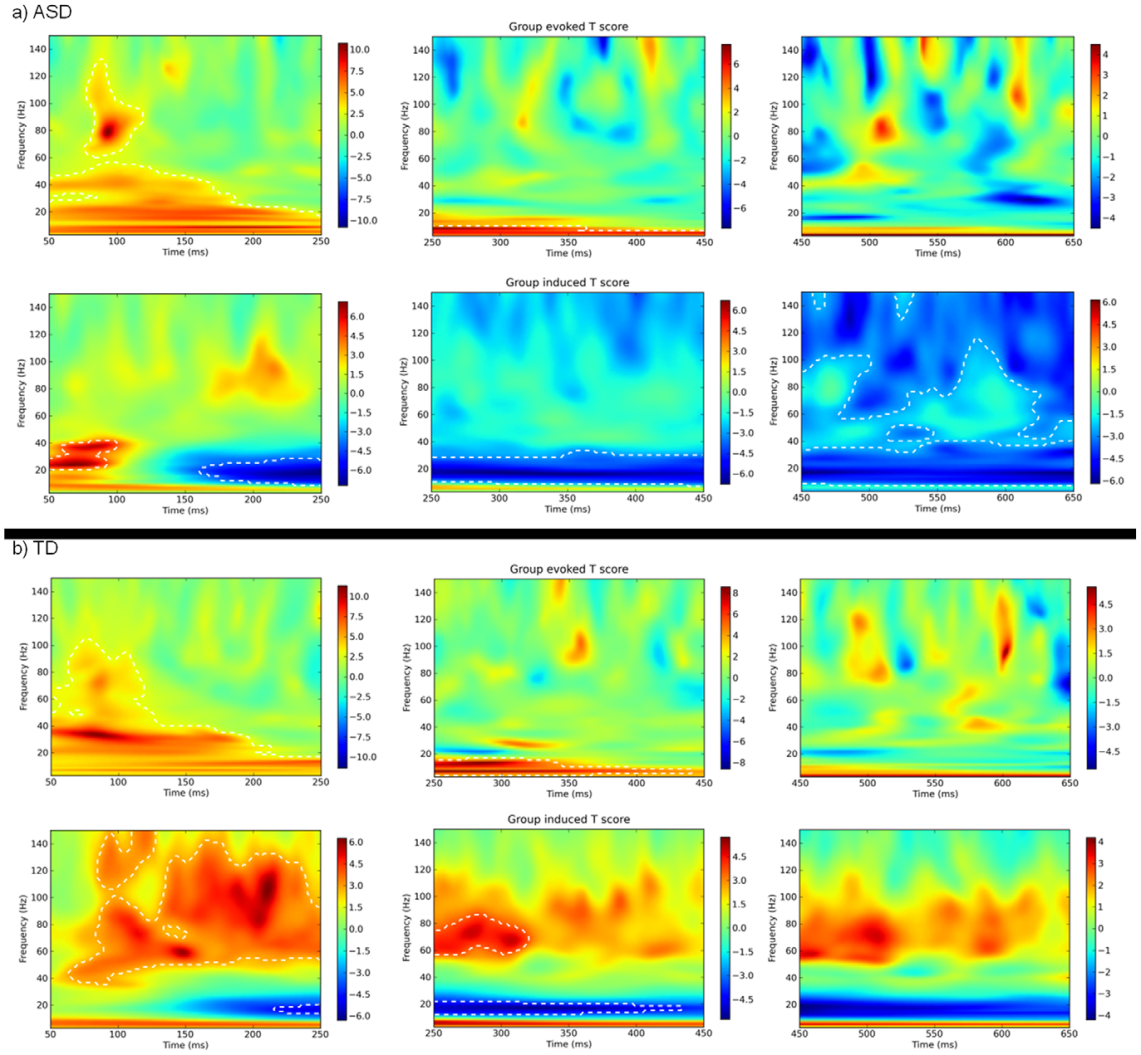
Virtual electrode responses in the left occipital pole (L-OP; −20, −92, 16) for ASD (a) and TD (b) participants. Evoked responses are displayed in the upper row of each figure; induced responses are displayed in the lower row. All responses indicate within subjects changes from baseline; changes significant at p<.05 level are indicated within dotted lines.

Figure 6 displays the virtual electrode response for the left occipital pole (L-OP). As in the right LOC site, both groups showed significant evoked responses in the first 50–250 ms but not after. Contrasting responses were observed for induced activity: ASD participants showed a short significant 20–40 Hz response between 50 and 100 ms, and then only reductions in power in the beta range (12–30 Hz). For TD participants beta-band decreases from 150 ms were again observable, but they were accompanied by significant increases in induced gamma activity from 80 ms post-stimulus.

#### Right fusiform gyrus

**Figure 7:**
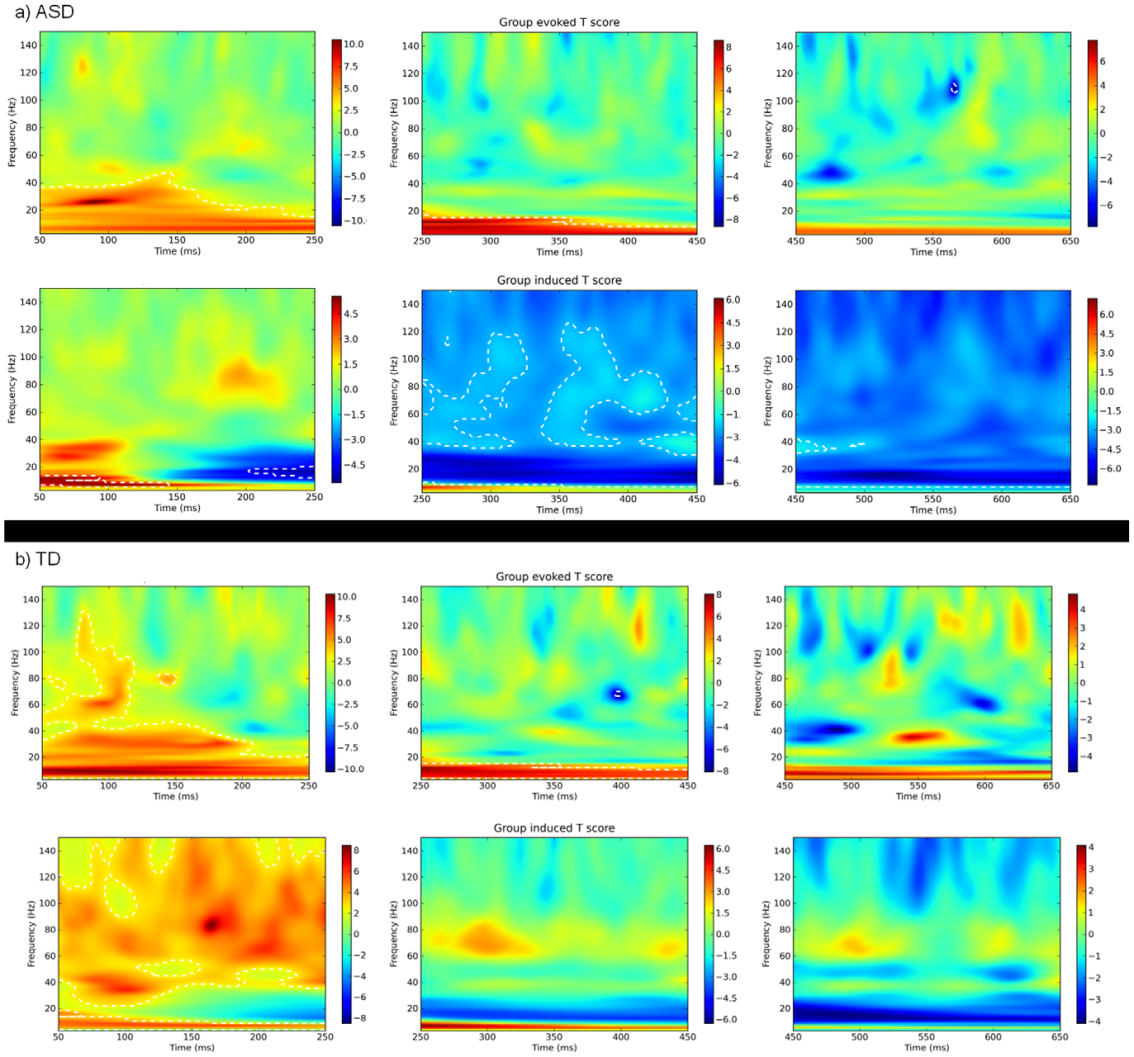
Virtual electrode responses in the right fusiform gyrus (R-FG; 32, −57, −3) for ASD (a) and TD (b) participants. Evoked responses are displayed in the upper row of each figure; induced responses are displayed in the lower row. All responses indicate within subjects changes from baseline; changes significant at p<.05 level are indicated within dotted lines.

Virtual electrode responses in the right fusiform gyrus (R-FG) are displayed for each group in figure 7. As in the L-OP, significant evoked responses were evident for both groups in the first 50–250 ms, peaking around 100 ms. The ASD response was constrained to 0–40 Hz and peaked around 25–30 Hz, whereas the control response extended to over 100 Hz. Other than a consistent response in the 0–15 Hz range, neither group indicated any other significant changes in evoked power for the remainder of the epoch. For induced responses, significant increases in power across the bandwidth were observed for control participants in the first 50–250 ms, with a clear 80 Hz peak around 160–170 ms. No such response was observed in the ASD group: increases in induced power were observed around 10 Hz for the first 150 ms, but following this significant decreases in power were seen in the beta range and then across the whole of the bandwidth. As in the right LOC and left OP, the ASD induced response was characterised by strong and widespread reduction of gamma oscillations from 150 ms onwards, although in the R-FG this appeared to extend across the bandwidth even earlier than in the L-OP. Whereas decreases in >30 Hz power were evident from 250 ms onwards in the R-FG, they were only significantly evident in the L-OP from 450 ms post stimulus onset.

### Specific Emotions

The responses for specific emotions were highly similar to the whole-head beamforming results for all emotions combined. The ASD response to individual emotions showed a much reduced gamma response: no significant increases in gamma power were observed for any emotion throughout the epoch. Significant *decreases* in gamma power were observed for anger (50–250 ms: −56, −20, 58, left precentral gyrus, *t* = −5.55) disgust, (250–450 ms: 30, 50, 44, right frontal pole, *t* = −5.97; 450–650 ms: −50, −20, 14, central opercular cortex, *t* = −5.40) and sadness (450–650 ms: left lateral occipital cortex, −36, −70, −44, *t* = −5.66). In contrast, significant decreases in lower-band power from 250 ms onwards were observed for all emotions in occipital pole and lateral occipital cortical areas (all voxels significant at *p*<.05).

In controls, significant increases in gamma-frequency power were observed in visual cortical regions from 50 ms onwards, specifically for areas of the occipital pole (disgust, fear, surprise) lingual gyrus (anger, fear, happiness, sadness, surprise) and lateral occipital cortex (all emotions; all voxels significant at *p*<.05). From 250 ms, decreases in lower-band power were also seen, centring primarily in the right occipital pole and left lateral occipital cortex (all emotions).

### Relation to AQ

Backwards-method regression analysis was used to assess how autistic behaviours related to the observed differences in gamma activation. As the peak between-group difference in gamma response was evident between 250–450 ms, the dependent variable used in the analysis was the mean t-score for each participant’s induced power response during this window, based on the time-frequency plots for virtual electrodes at the i) right LOC, ii) left OP and iii) right FG sites across all participants (ASD and TD combined). AQ, Age & FSIQ scores were included as predictors in the model.

For the right lateral occipital cortex site, a model including AQ only was returned (*R^2^* = .233, *F* (1, 22) = 6.689, *p* = .017). AQ negatively predicted t-scores for the induced gamma response (stan. *β* = −.483,*t* = −2.586, *p* = .017) indicating that higher scores for autistic behaviour were related to weaker induced gamma responses (compared to baseline). Age and IQ made no significant contribution (age: stan. *β* = .117, *t* = 0.606, *p* = .551; FSIQ: stan. *β* = .134, *t* = 0.690, *p* = .498). Very similar models were returned for predictors of the gamma response in the left occipital pole (*R^2^* = .212, *F* (1, 22) = 5.926, *p* = .023) and right fusiform gyrus (*R^2^* = .158, *F* (1, 22) = 4.131, *p* = .054). At both sites AQ was associated with gamma responses (Left OP: stan. *β* = −.461, *t* = −2.434, *p* = .023; Right FG: stan. *β* = −.398, *t* = −2.032, *p* = .054) but age and FSIQ were not (all stan. *β>*.140, all *t*<0.700, all *p*>.490).

For comparison the above analyses were also run for evoked gamma responses from the same window, although the predictive power of AQ was less clear across sites. In the right LOC, AQ predicted evoked responses (stan. *β* = −.410, *t* = −2.109, *p* = .047); in the left OP, evoked responses were predicted by AQ (stan. *β* = −.442, *t* = −2.365, *p* = .028) but also FSIQ (stan. *β* = −.352, *t* = −1.881, *p* = .074); while in the right FG no predictive relationship was observed between AQ and evoked responses (stan. *β* = −.115, *t* = −.533, *p* = .600, n.s.). When induced and evoked responses were directly compared for their ability to predict AQ, t-scores for evoked responses were only marginally retained for the right LOC (stan. *β* = −.316, *t* = −1.723, *p* = .100), whereas induced responses significantly predicted AQ in all three sites (all stan. *Β*>−.398, all *p*<.054). That is, the relationship between AQ and induced gamma responses was stronger than that for evoked responses across all the sites studied.

## Discussion

The main finding of the present study was evidence of specific abnormalities in the induced gamma response of ASD participants to emotional faces. Direct contrasts between the groups indicated that ASD and TD participants differed most significantly in the response of right lateral occipital areas to faces. When within-groups beamforming was used to localise sources of event-related activity in more detail, a strong gamma (30–80 Hz) response was observed in controls in visual areas, but this was absent in ASD participants. Control participants activated areas in the lateral occipital cortex, lingual gyrus and occipital fusiform gyrus throughout the time course in response to faces. In contrast, the only significant changes observed for ASD participants were specific power reductions in the left supramarginal gyrus and left precentral gyrus from 250–450 ms. No differences were seen in the lower frequency band (3–30 Hz).

Time-frequency analysis of the virtual electrodes supported and added to this picture. The initial evoked responses to faces in the right lateral occipital cortex, left occipital pole and right fusiform gyrus were similar for both groups, with increases in power peaking between 50 and 100 ms. The groups diverged, however, after this response. From 150 ms, ASD participants displayed strongly inhibited oscillatory activity in the beta band (12–30 Hz) before displaying decreases in induced power across the gamma range. At the same time, control participants showed increases in the induced gamma response. In the right lateral occipital cortex and fusiform gyrus, this occurred in the earliest time window examined (50–250 ms) but in the left occipital pole area this activity was maintained throughout the time course. So while control participants were increasing and maintaining induced gamma-band activity, ASD participants’ gamma response was decreasing. This pattern was evident in both the data-driven and theory-driven virtual electrode sites and it related to scores on the AQ: participants with higher AQ produced weaker induced gamma responses.

The presence of abnormalities in the early visual response to faces in ASD participants is consistent with previous neurophysiological research [3]. Using MEG, Bailey et al [26] reported reduced responses to faces in ASD adults at 145 ms centring on right inferior occipital-temporal cortex, alongside group differences as early as 30–60 ms in right anterior temporal regions (see also [29]). Kylliainen et al [32] also using MEG, reported differences in the processing of faces and other complex objects (motorbikes) at 100 ms in ASD children matched with typically-developing controls.

What the present study adds to these findings is evidence of irregular modulation of induced gamma-band activity from 150 ms, apparent in both primary visual cortical areas and the right fusiform gyrus, in young people with ASD. The presence of induced gamma abnormalities but intact evoked responses is consistent with the findings of Grice et al [41] concerning upright and inverted faces and Brown et al [50] for Kanisza stimuli, but contrasts with the results of Stroganova et al [53] and previous research on auditory responses in ASD [43], [44], [55]. Based on this pattern, it seems likely that problems with gamma functioning vary across visual and auditory modalities in autism.

From a cognitive viewpoint, these findings support the suggestion that ASD individuals may have difficulty processing faces in a holistic way [7] and that such differences occur early in the processing stream [73]. At this stage it is not clear why these abnormalities occur, but they may underlie the commonly described local bias or weak central coherence in ASD cognitive processing [51], [52]. The predictive relation between AQ and induced gamma observed here suggests that visual processing abnormalities are related to autistic tendencies and behaviour in a meaningful sense, although it does not show that differences in the response to face stimuli lead to more complex difficulties with social cognition and function. As noted by Behrmann et al [74], the presence of visual processing differences, independent of problems with social cognition, raises its own set of challenges for understanding autism. This has consequences both clinically and educationally as materials, interventions and services need to be shaped with sensory processing differences in mind [75].

However, caution should be taken in the interpretation of these results for a number of reasons, and these considerations map out questions for future research.

### Limitations

Firstly, the exact role of gamma oscillations in cortical processing is still a matter of considerable debate. Consistent with the hypothesis that distal functional connectivity is impaired in autism [48], [76], [77]. Gruber [78] has argued that induced gamma is related to synchronous, long-range cortical activity, while evoked activity is more constrained to the immediate sensory response. However, induced gamma oscillations have also been related to more basic aspects of sensory processing, such as luminance and contrast [79] and higher cognitive functions, such as attention and memory [80], [81]. More work is needed on the separate functions of evoked and induced gamma before disruptions in the ASD response can be fully understood.

Second is the issue of clinical specificity. As noted by others [43], [44] gamma abnormalities have been reported in relation to a range of processes in individuals with schizophrenia [82] and irregular modulation of gamma oscillations have also been observed in children with ADHD [83] and Williams Syndrome [41]. Moreover, the generation of induced gamma activity across healthy individuals may vary considerably [84]. This suggests that though gamma abnormalities may be an important avenue for further research in autism, they are unlikely to provide a specific biomarker for autism. Instead, further research may shed light on different characteristics of ASD sensory processing, and how these may be remediated [85].

Thirdly, other methodological limitations of the present study need to be considered. When assessing face processing in autism, it is important to control for viewing behaviours; brain activation could be different because, for example, children are looking in a different place [86]. We asked participants to look at a fixation cross between trials, we carried out continuous videoing of participants faces throughout scanning, and there were no obvious differences between groups, with participants in both attending intently throughout. Indeed ASD participants followed instructions very closely. Furthermore, the presence of intact evoked responses in ASD participants strongly suggests that participants were still viewing the stimuli throughout the scan. Nevertheless, an important follow-up to this research would be to integrate analysis of the gamma response to faces with eye-tracking methods, to assess areas of facial attention in ASD participants, potential differences in viewing strategies, and how the two relate to local versus global stimulus perception. Atypical viewing behaviours, such as attending more to the mouth rather than eyes when viewing faces, have been reported in ASD individuals and their relatives [87]–[89] and appear to relate to activation of the fusiform gyrus and the amygdala [70], [90]. A key question to answer would be whether or not induced gamma abnormalities persist even when ASD individuals are encouraged to employ more regular viewing behaviours.

One further issue is that a relatively broad age range was used in the study. The regression analysis did not indicate any significant influence of age on induced gamma responses but follow-up studies could use stricter age banding, now that this feasibility work has shown these techniques can be used across a range of ages.

### Future Directions

Future studies should include a) comparisons with matched controls from different clinical groups (e.g. specific language impairment, ADHD etc) b) use of eye-tracking to examine differences in visual attention and processing strategies, and how these relate to apparent gamma abnormalities, and c) replication with a more restricted age range or cross-sectional samples. Future work could also explore whether gamma band abnormalities are different when comparing face recognition alone compared to emotion face recognition. Finally, further work comparing gamma responses for faces and other complex visual objects (such as buildings) in ASD participants is necessary to clarify the role and specificity of gamma abnormalities in autism.

### Conclusion

The present study piloted the use of beamforming techniques in MEG to examine the responses of children and young people with ASD to emotions on faces. In marked contrast to controls, ASD participants did not produce induced gamma oscillations in the 30–80 Hz range when viewing faces, across all types of emotion. Evoked gamma responses and responses in other bands were largely intact. This is consistent with hypotheses about the role of induced gamma in feature binding and the proposed disruption to such binding in autism, suggesting a possible mechanism for difficulties in face and emotion processing in ASD individuals. However, further work is needed on the specific processing roles of gamma oscillations and the clinical specificity of gamma disruption. A better understanding of specific neuronal dysfunction will ultimately link to the recent advances in developmental research, neuropsychology and brain physiology, leading to a more rounded understanding of autism.

## References

[1] World Health Organisation (1993) The ICD –10 Classification of Mental and Behavioural Disorders:- Diagnostic Criteria for Research: General World Health Organisation.

[2] Weeks S, Hobson RP (1987) The salience of facial expression for autistic children. Journal of Child Psychology and Psychiatry 28 (1): 137–151. 10.1111/j.1469-7610.1987.tb00658.x 3558531

[3] Dawson G, Webb SJ, McPartland J (2005) Understanding the nature of face processing impairment in autism: insights from behavioral and electrophysiological studies. Developmental Neuropsychology 27 (3): 403–424. 10.1207/s15326942dn2703_6 15843104

[4] Harms M, Martin A, Wallace G (2010) Facial emotion recognition in autism spectrum disorders: a review of behavioral and neuroimaging studies. Neuropsychology Review 20: 290–322. 20809200 10.1007/s11065-010-9138-6

[5] Jemel B, Mottron L, Dawson M (2006) Impaired face processing in autism: Fact or artifact? Journal of Autism and Developmental Disorders 36: 91–106. 16477517 10.1007/s10803-005-0050-5

[6] Tracy J, Robins R, Schriber R, Solomon M (2011) Is emotion recognition impaired in individuals with Autism Spectrum Disorders? Journal of Autism and Developmental Disorders 41(1): 102–109. 20464465 10.1007/s10803-010-1030-y PMC3005106

[7] Gross T (2005) Global-local precedence in the perception of facial age and emotional expression by children with autism and other developmental disabilities. Journal of Autism and Developmental Disorders 35: 773–785. 16283086 10.1007/s10803-005-0023-8

[8] Humphreys K, Minshew N, Leonard GL, Behrmann M (2007) A fine-grained analysis of facial expression processing in high-functioning adults with autism. Neuropsychologia 45 (4): 685–695. 10.1016/j.neuropsychologia.2006.08.003 17010395

[9] Rump K, Giovannelli J, Minshew N, Strauss M (2009) The Development of Emotion Recognition in Individuals With Autism. Child Development 80 (5): 1434–1447. 10.1111/j.1467-8624.2009.01343.x PMC3085906 19765010

[10] Ashwin C, Chapman E, Colle L, Baron-Cohen S (2006) Impaired recognition of negative basic emotions in autism: a test of the amygdala theory. Social Neuroscience, 1 (3–4): 349–363. 10.1080/17470910601040772 18633799

[11] Pelphrey K, Sasson N, Reznick J, Paul G, Goldman B, et al (2002) Visual scanning of faces in autism. Journal of Autism and Developmental Disorders, 32 (4): 249–261. 10.1023/a:1016374617369 12199131

[12] Wright B, Clarke N, Jordan J, Young A, Clarke P, et al (2008) Emotion recognition in faces and the use of visual context in young people with high-functioning autism spectrum disorders. Autism 12 (6): 607–626. 10.1177/1362361308097118 19005031

[13] Golarai G, Grill-Spector K, Reiss AL (2006) Autism and the development of face processing. Clinical Neuroscience Research, 6 (3–4): 145–160. 10.1016/j.cnr.2006.08.001 PMC2174902 18176635

[14] Critchley H, Daly E, Bullmore E, Williams S, Van Amelsvoort T, et al (2000) The functional neuroanatomy of social behaviour: changes in cerebral blood flow when people with autistic disorder process facial expressions. Brain, 123 (11): 2203–2212. 10.1093/brain/123.11.2203 11050021

[15] Deeley Q, Daly E, Surguladze S, Page L, Toal F, et al (2007) An event related functional magnetic resonance imaging study of facial emotion processing in Asperger syndrome. *Biollogical Psychiatry*, 62 (3): 207–217. 10.1016/j.biopsych.2006.09.037 17400195

[16] Humphreys K, Hasson U, Avidan G, Minshew N, Behrmann M (2008) Cortical patterns of category-selective activation for faces places and objects in adults with autism. Autism Research 1 (1): 52–63. 19360650 10.1002/aur.1 PMC2765685

[17] Schultz R, Gauthier I, Klin A, Fulbright R, Anderson A, et al (2000) Abnormal ventral temporal cortical activity during face discrimination among individuals with autism and Asperger syndrome. Archives of General Psychiatry 57 (4): 331–340. 10768694 10.1001/archpsyc.57.4.331

[18] Hadjikhani N, Joseph R, Snyder J, Chabris C, Clark J, et al (2004) Activation of the fusiform gyrus when individuals with autism spectrum disorder view faces. Neuroimage, 22 (3): 1141–1150. 15219586 10.1016/j.neuroimage.2004.03.025

[19] Pierce K, Redcay E (2008) Fusiform function in children with an autism spectrum disorder is a matter of “who”. *Biol Psychiatry*, 64 (7): 552–560. 18621359 10.1016/j.biopsych.2008.05.013 PMC2673799

[20] Pierce K, Muller R, Ambrose J, Allen G, Courchesne E (2001) Face processing occurs outside the fusiform ‘face area’ in autism: evidence from functional MRI. Brain 124 (10): 2059–2073. 11571222 10.1093/brain/124.10.2059

[21] Kleinhans N, Richards T, Sterling L, Stegbauer K, Mahurin R, et al (2008) Abnormal functional connectivity in autism spectrum disorders during face processing. Brain, 131 (4): 1000–1012. 18234695 10.1093/brain/awm334

[22] Koshino H, Kana R, Keller T, Cherkassky V, Minshew N, et al (2008) FMRI investigation of working memory for faces in autism: Visual coding and underconnectivity with frontal areas. Cerebral Cortex, 18 (2): 289–300. 17517680 10.1093/cercor/bhm054 PMC4500154

[23] Wicker B, Fonlupt P, Hubert B, Tardif C, Gepner B, et al (2008) Abnormal cerebral effective connectivity during explicit emotional processing in adults with autism spectrum disorder. Social Cognitive and Affective Neuroscience, 3 (2): 135–143. 19015104 10.1093/scan/nsn007 PMC2555468

[24] Monk C, Weng S, Wiggins J, Hurapati N, Louro H, et al (2010) Neural circuitry of emotional face processing in autism spectrum disorders. *Journal of Psychiatry and Neuroscience*, 35 (2): 105–114. 20184808 10.1503/jpn.090085 PMC2834792

[25] Scherf S, Luna B, Minshew N, Behrmann M (2010) Location, location, location: alterations in the functional topography of face- but not object- or place-related cortex in adolescents with autism. *Frontiers in Human Neuroscience*, 4 (26): 1–16. 20631857 10.3389/fnhum.2010.00026 PMC2904054

[26] Bailey AJ, Braeutigam S, Jousmaki V, Swithenby SJ (2005) Abnormal activation of face processing systems at early and intermediate latency in individuals with autism spectrum disorder: a magnetoencephalographic study. European Journal of Neuroscience 21 (9): 2575–2585. 15932615 10.1111/j.1460-9568.2005.04061.x

[27] O’Connor K, Hamm J, Kirk I (2007) Neurophysiological responses to face, facial regions and objects in adults with Asperger’s syndrome: an ERP investigation. International Journal of Psychophysiology, 63 (3): 283–293. 17267061 10.1016/j.ijpsycho.2006.12.001

[28] Wong T, Fung P, Chua S, McAlonan G (2008) Abnormal spatiotemporal processing of emotional facial expressions in childhood autism: dipole source analysis of event-related potentials.European Journal of Neuroscience. 28 (2): 407–416. 10.1111/j.1460-9568.2008.06328.x 18702712

[29] Bentin S, Allison T, Puce A, Perez E, McCarthy G (1996) Electrophysiological studies of face perception in humans. Journal of Cognitive Neuroscience, 8 (6): 551–565. 20740065 10.1162/jocn.1996.8.6.551 PMC2927138

[30] McPartland J, Dawson G, Webb S, Panagiotides H, Carver L (2004) Event-related brain potentials reveal anomalies in temporal processing of faces in autism spectrum disorder. Journal of Child Psychology and Psychiatry 45 (7): 1235–1245. 15335344 10.1111/j.1469-7610.2004.00318.x

[31] Kylliainen A, Braeutigam S, Hietanen J, Swithenby S, Bailey A (2006) Face- and gaze-sensitive neural responses in children with autism: a magnetoencephalographic study, European Journal of Neuroscience 24. (9): 2679–2690. 10.1111/j.1460-9568.2006.05132.x 17100856

[32] Van Veen B, van Drongelen W, Yuchtman M, Suzuki A (1997) Localization of brain electrical activity via linearly constrained minimum variance spatial filtering. IEEE Transactions on Biomedical Engineering 44 (9): 867–880. 9282479 10.1109/10.623056

[33] Robinson S, Vrba J (1999) Functional neuroimaging by Synthetic Aperture Magnetometry (SAM) Tohoku Univ, Press: Japan, pp. 302–305.

[34] Huang M, Shih J, Lee R, Harrington D, Thoma R, et al (2004) Commonalities and differences among vectorized beamformers in electromagnetic source imaging. Brain Topography 16: 139–158. 15162912 10.1023/b:brat.0000019183.92439.51

[35] Lee L, Andrews T, Johnson S, Woods W, Gouws A, et al (2010) Neural responses to rigidly moving faces displaying shifts in social attention investigated with fMRI and MEG. Neuropsychologia 48: 477–490. 19833143 10.1016/j.neuropsychologia.2009.10.005

[36] Maratos F, Anderson S, Hillebrand A, Singh K, Barnes G (2007) The spatial distribution and temporal dynamics of brain regions activated during the perception of object and non-object patterns. Neuroimage 34: 371–383. 17055298 10.1016/j.neuroimage.2006.09.017

[37] Millman R, Prendergast G, Kitterick P, Woods W, Green G (2010) Spatiotemporal reconstruction of the auditory steady-state response to frequency modulation using magnetoencephalography. Neuroimage 49: 745–758. 19699806 10.1016/j.neuroimage.2009.08.029

[38] Muthukumaraswamy S, Singh K (2008) Spatiotemporal frequency tuning of BOLD and gamma band MEG responses compared in primary visual cortex. Neuroimage 40: 1552–1560. 18337125 10.1016/j.neuroimage.2008.01.052

[39] Honaga E, Ishii R, Kurimoto R, Canuet L, Ikezawa K, et al (2010) Post movement beta rebound abnormality as indicator of mirror neuron system dysfunction in autism spectrum discorder: An MEG study. Neuroscience Letters 478(3): 141–145. 20452402 10.1016/j.neulet.2010.05.004

[40] Grice S, Spratling M, Karmiloff-Smith A, Halit H, Csibra G, et al (2001) Disordered visual processing and oscillatory brain activity in autism and Williams syndrome. Neuroreport 12 (12): 2697–2700. 10.1097/00001756-200108280-00021 11522950

[41] Orekhova E, Stroganova T, Nygren G, Tsetlin M, Posikera I, et al (2007) Excess of high frequency electroencephalogram oscillations in boys with autism. Biological Psychiatry 62: 1022–1029. 17543897 10.1016/j.biopsych.2006.12.029

[42] Wilson T, Rojas D, Reite M, Teale P, Rogers S (2007) Children and adolescents with autism exhibit reduced MEG steady-state gamma responses. Biological Psychiatry 62 (3): 192–197. 16950225 10.1016/j.biopsych.2006.07.002 PMC2692734

[43] Rojas D, Maharajh K, Teale P, Rogers S (2008) Reduced neural synchronization of gamma-band MEG oscillations in first-degree relatives of children with autism. BMC Psychiatry 8. (66). 10.1186/1471-244X-8-66 PMC2518921 18673566

[44] Tallon-Baudry C, Bertrand O (1999) Oscillatory gamma activity in humans and its role in object representation. Trends in Cognitive Sciences 3 (4): 151–162. 10322469 10.1016/s1364-6613(99)01299-1

[45] Varela F, Lachaux J, Rodriguez E, Martinerie J (2001) The brainweb: phase synchronization and large-scale integration. Nature Reviews Neuroscience 2: 229–239. 11283746 10.1038/35067550

[46] Kaiser J, Lutzenburger W (2003) Induced gamma-band activity and human brain function. *The Neuroscientist* 9: 475–484. 14678580 10.1177/1073858403259137

[47] Brock J, Brown C, Boucher J, Rippon G (2002) The temporal binding deficit hypothesis of autism. Development and Psychopathology 14: 209–224. 12030688 10.1017/s0954579402002018

[48] Pulvermuller F, Birbaumer N, Lutzenburger B, Mohr B (1997) High-frequency brain activity: Its possible role in attention, perception and language processing. Progress inNeurobiology 52 (5): 427–445. 10.1016/s0301-0082(97)00023-3 9304700

[49] Brown C, Gruber T, Boucher J, Rippon G, Brock J (2005) Gamma abnormalities during perception of illusory figures in autism. *Cortex*, 41 (3): 364–. 15871601 10.1016/s0010-9452(08)70273-9

[50] Happe F, Frith U (2006) The weak coherence account: detail-focused cognitive style in autism spectrum disorders. Journal of Autism and Developmental Disorders 36: 5–25. 16450045 10.1007/s10803-005-0039-0

[51] Mottron L, Dawson M, Soulieres I, Hubert B, Burack J (2006) Enhanced perceptual functioning in autism: An update and eight principles of autistic perception. Journal of Autism and Developmental Disorders 36: 27–43. 16453071 10.1007/s10803-005-0040-7

[52] Stroganova TA, Orekhova EV, Prokofyev AO, Tsetlin MM, Gratchev VV, et al. (2011) High-frequency oscillatory response to illusory contour in typically developing boys and boys with autism spectrum disorders. *Cortex*, In press. http://dx.doi.org/10.1016/j.cortex.2011.02.016. 10.1016/j.cortex.2011.02.016 21458787

[53] Rojas D, Teale P, Maharajh K, Kronberg E, Youngpeter K, e al (2011) Transient and steady-state auditory gamma-band responses in first-degree relatives of people with autism spectrum disorder. Molecular Autism 2: 11 doi:10.1186/2040-2392-2-11. 21729257 10.1186/2040-2392-2-11 PMC3143088

[54] Gandal M, Edgar C, Ehrlichman R, Mehta M, Roberts T, et al (2011) Validating Gamma Oscillations and Delayed Auditory Responses as Translational Biomarkers of Autism. Biological Psychiatry 68: 1100–1106. 10.1016/j.biopsych.2010.09.031 PMC5070466 21130222

[55] Lord C, Rutter M, Le Couteur A (1994) Autism Diagnostic Interview-Revised: A version of a diagnostic interview for caregivers of individuals with possible pervasive developmental disorders, Journal of Autism and Developmental Disorders. 25: 659–685. 10.1007/BF02172145 7814313

[56] Lord C, Risi S, Lambrecht L, Cook E, Leventhal B, et al (2000) The Autism Diagnostic Observation Schedule–Generic: A standard measure of social and communication deficits associated with the spectrum of autism. Journal of Autism and Developmental Disorders 30: 205–223. 11055457

[57] Baron-Cohen S, Hoekstra R, Knickmeyer R, Wheelwright S (2006) The Autism-Spectrum Quotient (AQ) – Adolescent Version. Journal of Autism and Developmental Disorders 36: 343–350. 16552625 10.1007/s10803-006-0073-6

[58] Wechsler D (1999) WASI: Wechsler Abbreviated Scales of Intelligence, San Antonio TX: The Psychological Corporation.

[59] Ekman P, Friesen W (2006) Pictures of facial affect, Consulting Psychologists Press: Palo Alto CA.

[60] Young AW, Perrett DI, Calder AJ, Spengelmeyer R, Ekman P (2002) Facial Expressions of Emotion Stimuli and Tests (FEEST) Bury St Edmunds: Thames Valley Test Company.

[61] Johnson R, Hirschkoff E, Buchanan D (2003) Full-sensitivity biomagnetometers: Sam Williamson’s vision brought to life. In Z. Lu & L. Kaufman (Eds.), Magnetic source imaging of the human brain (pp. 217–229). NJ: Lawrence Erlbaum.

[62] Kozinska D, Carducci F, Nowinski K (2001) Automatic alignment of EEG/MEG and MRI data sets. Clinical Neurophysiology 112 (8): 1553–1561. 10.1016/s1388-2457(01)00556-9 11459696

[63] Hymers M, Prendergast G, Johnson S, Green G (2009) Source stability index: A novel beamforming based localisation metric. Neuroimage 49: 1385–1397. 19800010 10.1016/j.neuroimage.2009.09.055

[64] Vrba J, Robinson S (2001) Signal processing in magnetoencephalography. Methods 25 (2): 249–271. 10.1006/meth.2001.1238 11812209

[65] Singh K, Barnes G, Hillebrand A (2003) Group imaging of task-related changes in cortical synchronisation using non-parametric permutation testing. *Neuroimage, 19* (4): 1589–1601. 10.1016/s1053-8119(03)00249-0 12948714

[66] Prendergast G, Johnson S, Hymers M/, Woods W, Green G (2011) Non-parametric statistical thresholding of baseline free MEG beamformer images. NeuroImage 54 (2): 906–918. 10.1016/j.neuroimage.2010.08.005 20696257

[67] Nichols T, Holmes A (2002) Nonparametric permutation tests for functional neuroimaging: a primer with examples. Human Brain Mapping 15 (1): 1–25. 10.1002/hbm.1058 PMC6871862 11747097

[68] Kanwisher N, McDermott J, Chun MM (1997) The fusiform face area: a module in human extrastriate cortex specialized for face perception. The Journal of Neuroscience 17(11): 4302. 9151747 10.1523/JNEUROSCI.17-11-04302.1997 PMC6573547

[69] Ashwin C, Baron-Cohen S, Wheelwright S, O’Riordan M, Bullmore ET (2007) Differential activation of the amygdala and the ‘social brain’ during fearful face-processing in Asperger Syndrome, Neuropsychologia 45. (1): 2–14. 10.1016/j.neuropsychologia.2006.04.014 16806312

[70] Dalton K, Nacewicz B, Johnstone T, Schaefer H, Gernsbacher M, et al (2005) Gaze fixation and the neural circuitry of face processing in autism, Nature Neuroscience 8. (4): 519–526. 10.1038/nn1421 PMC4337787 15750588

[71] Stockwell RG, Mansinha L, Lowe RP (1996) Localization of the complex spectrum: The S-Transform. IEEE Trans Sig Proc 44 (4): 998–1001.

[72] Wheat K, Cornelissen P, Frost S, Hanse P (2010) During visual word recognition phonology is accessed within 100 ms and may be mediated by a speech production code: evidence from magnetoencephalography. Journal of Neuroscience 30 (15): 5229–5233. 10.1523/JNEUROSCI.4448-09.2010 PMC3419470 20392945

[73] Batty M, Meaux E, Wittemeyer K, Roge B, Taylor M (2011) Early processing of emotional faces in children with autism: An event-related potential study. Journal of Experimental Child Psychology, 109 (4): 430–. 21458825 10.1016/j.jecp.2011.02.001

[74] Behrmann M, Thiomas C, Humphreys K (2006) Seeing it differently: visual processing in autism. Trends in Cognitive Sciences 10 (6): 258–264. 16713326 10.1016/j.tics.2006.05.001

[75] Baranek (2002) *Efficacy of Sensory and Motor Interventions for Children with Autism, Journal of Autism and Developmental Disorders,* 32, 397–422. 10.1023/a:1020541906063 12463517

[76] Belmonte M, Cook E, Anderson G, Rubenstein J, Greenough W, et al (2004) Autism as a disorder of neural information processing:directions for research and targets for therapy. Molecular Psychiatry 9: 646–663. 15037868 10.1038/sj.mp.4001499

[77] Just M, Cherkassky V, Keller T, Minshew N (2004) Cortical activation and synchronization during sentence comprehension in high-functioning autism: evidence of underconnectivity. Brain 127: 1811–1821. 15215213 10.1093/brain/awh199

[78] Gruber T (2008) Sources of synchronized induced gamma-band responses during a simple object recognition task: A replication study in human MEG. Brain Research 1196: 74–84. 18234156 10.1016/j.brainres.2007.12.037

[79] Adjamian P, Hadjipapas A, Barnes G, Hillebrand A, Holliday I (2008) Induced gamma activity in primary visual cortex is related to luminance and not color contrast: a MEG study, Journal of Vision 8. (7): 1–7. 10.1167/8.7.4 19146237

[80] Herrmann C, Munk M, Engel A (2004) Cognitive functions of gamma band activity. Trends in Cognitive Sciences 8: 347–355. 15335461 10.1016/j.tics.2004.06.006

[81] Jensen O, Kaiser J, Lachaux J (2007) Human gamma frequency oscillations with attention and memory.Trends in Neurosciences. 30: 317–324. 10.1016/j.tins.2007.05.001 17499860

[82] Williams S, Boksa P (2010) Gamma oscillations and schizophrenia. Journal of Psychiatry and Neuroscience 35 (2): 75–77. 20184803 10.1503/jpn.100021 PMC2834788

[83] Lenz D, Krauel K, Flechtner H, Schadow J, Hinrichs H, et al (2010) Altered evoked gamma-band responses reveal impaired early visual processing in ADHD children. Neuropsychologia 48: 1985–1993. 20350556 10.1016/j.neuropsychologia.2010.03.019

[84] Muthukumaraswamy S, Singh K, Swettenham J, Jones D (2009) Visual gamma oscillations and evoked responses: Variability repeatability and structural MRI correlates. Neuroimage 49: 3349–3357. 19944770 10.1016/j.neuroimage.2009.11.045

[85] Sokhadze E, El-Baz A, Baruth J, Mathai G, Sears L, et al (2009) Effects of Low Frequency Repetitive Transcranial Magnetic Stimulation (rTMS) on Gamma Frequency Oscillations and Event-Related Potentials During Processing of Illusory Figures in Autism. Journal of Autism and Developmental Disorders 39: 619–634. 19030976 10.1007/s10803-008-0662-7

[86] Klin A (2008) Three things to remember if you are a functional magnetic resonance imaging researcher of face processing in autism spectrum disorders. Biological Psychiatry 64: 549–551. 18778978 10.1016/j.biopsych.2008.07.028 PMC2596985

[87] Adolphs R, Spezio M, Parlier M, Piven J (2008) Distinct Face-Processing Strategies in Parents of Autistic Children. Current Biology 18: 1090–1093. 18635351 10.1016/j.cub.2008.06.073 PMC2504759

[88] Joseph R, Tanaka J (2002) Holistic and part-based face recognition in children with autism. Journal of Child Psychology & Psychiatry, 43: 8, 1–14. 10.1111/1469-7610.00142 12751845

[89] Spezio M, Adolphs R, Hurley R, Piven J (2007) Abnormal use of facial information in high-functioning autism. Journal of Autism and Developmental Disorders 37: 929–939. 17006775 10.1007/s10803-006-0232-9

[90] Dalton K, Nacewicz B, Alexander A, Davidson R (2007) Gaze-Fixation, Brain Activation, and Amygdala Volume in Unaffected Siblings of Individuals with Autism. Biological Psychiatry 61: 512–520. 17069771 10.1016/j.biopsych.2006.05.019

